# Up-regulated mRNA expression of *VEGFA* receptors (*FLT1* and *KDR*) in placentas after assisted reproductive technology fertilization

**DOI:** 10.1007/s13353-023-00823-2

**Published:** 2024-01-18

**Authors:** Aleksandra E. Mrozikiewicz, Grażyna Kurzawińska, Michał Walczak, Marzena Skrzypczak-Zielińska, Marcin Ożarowski, Piotr Jędrzejczak

**Affiliations:** 1 Department of Obstetrics and Women’s Diseases, Poznan University of Medical Sciences, Polna 33, 60-535 Poznan, Poland; 2 Division of Perinatology and Women’s Diseases, Poznan University of Medical Sciences, Polna 33, 60-535 Poznan, Poland; 3 Laboratory of Molecular Biology in Division of Perinatology and Women’s Diseases, Poznan University of Medical Sciences, Polna 33, 60-535 Poznan, Poland; 4 Institute of Human Genetics, Polish Academy of Sciences, Strzeszyńska 32, 60-479 Poznan, Poland; 5 Department of Biotechnology, Institute of Natural Fibres and Medicinal Plants, National Research Institute, 60-630 Poznan, Poland; 6 Department of Infertility and Reproductive Endocrinology, Poznan University of Medical Sciences, Polna 33, 60-535 Poznan, Poland

**Keywords:** ART, IVF, Placental expression, VEGFA, FGF2, KDR, FLT1

## Abstract

Placental angiogenesis is a pivotal process for feto-maternal circulation and ensures efficient development of the placenta throughout pregnancy. Many factors during in vitro fertilization and embryo transfer procedures may affect placental gene expression and fetus development. The present study aimed to identify differences in angiogenesis-related gene (*VEGFA*, *FGF2*, *FLT1*, and *KDR*) expression profiles in placentas after assisted reproductive technology fertilization and natural conception in healthy women. In a case-control study, term placentas were collected from Caucasian women after assisted reproductive technology fertilization (*N* = 20) and after natural conception in women with uncomplicated pregnancy (*N* = 9). The mRNA expression in placentas was examined for *VEGFA*, *FGF2*, *FLT1*, and *KDR* genes by real-time quantitative polymerase chain reaction (RT-qPCR). Group stratification was performed for comparison of investigated genes between the type of embryo transferred (fresh/frozen), place of tissue donation (center/margin), and newborns’ gender (male/female). In the ART placentas, significant down-regulation of *VEGFA* gene (*p* = 0.016) and up-regulation of *FLT1* (*p* = 0.026) and *KDR* (*p* < 0.001) gene receptors were observed. Genes encoding *VEGFA* receptors were up-regulated in both fresh (ET) and frozen (FET) embryo transfer groups compared to controls. For the *FLT1* gene, a statistically significant difference was observed between the frozen embryo transfer group and the controls (*p* = 0.032). Relative expression of *KDR* was significantly higher for both embryo transfer groups compared to controls (*p* < 0.001) and between ET and FET (*p* = 0.002). No statistically significant differences were observed between placental expression in different places of tissue donation and newborns’ gender. We observed differences in the placental expression of *VEGFA* and its receptors *FLT1* and *KDR* in pregnancies after assisted reproductive technology compared to naturally conceived pregnancies. More research is needed to clarify these alterations that may affect placental development and fetal health.

## Introduction

Assisted reproductive technology (ART) has developed rapidly in recent years to support infertility treatment (Chen et al. 2023). This technology is utilized during a critical period of gamete and embryo development at the time when the genome is undergoing significant epigenetic remodeling, and changes in the environment can easily affect normal developmental programming (Heber and Ptak 2021; Chen et al. 2023).

Embryo implantation is a multistage process, conditioned by the action of several embryonic and maternal factors. The diverse and delicate cascade of overlapping molecular mechanisms makes it possible to achieve a balance of endocrine and auto- and paracrine factors and create a critical implantation window. During this period, the unique expression of biological molecules allows for proper embryo-endometrial balance and thus implantation of the embryo through signaling, localization, attachment, and invasion (Massimiani et al. 2019). During pregnancy, appropriate vascularization of the placenta is crucial for adequate maternal-fetal exchange. A number of factors including the endometrial receptor, decidual signaling, and immune systems are involved in the implantation process. Undoubtedly, cytokines and growth factors play an important role, modulating the angiogenetic process, which determines the formation of a receptive endometrium and then the proper development of the chorion and placenta. Proper vasculogenesis and angiogenesis, in turn, promote proper embryogenesis, as well as proper placental function (Bhattacharjee et al. 2021). This process is controlled by prostaglandins and the family of vascular endothelial growth factor (VEGF) (Hoozemans et al. 2004; Craciunas et al. 2019; Hernández-Vargas et al. 2020). In addition, trophoblast cells secrete large amounts of soluble receptors capable of binding both growth factors, affecting their bioavailability (Huppertz and Peeters 2005; Burton et al. 2009).

A broad spectrum of locally produced angiogenic factors has been identified in human placenta. The most potent angiogenic factors to promote vasculogenesis and angiogenesis in the placenta include VEGF family molecules, FGF family molecules, and the angiopoietin/Tie system (Wang and Zhao 2010). The most important angiogenic factors from the VEGF family are VEGF, placental growth factor (PlGF), and their receptors, VEGFR1/FLT1 and VEGFR2/KDR (Ahmed et al. 2000). VEGF and its receptor proteins are involved in the adaptation and modification of placental vessels. These processes include the regulation of vascular expansion and permeability and the protection of the vascular endothelium. In this way, the interactions between VEGF/PlGF and their receptors are crucial for proper angiogenesis and vasculogenesis (Ferrara et al. 2003).

The imbalance between the angiogenic factors of the VEGF family is associated with the development of pregnancy complications, such as spontaneous abortion, preterm delivery, gestational diabetes mellitus, gestational hypertension, preeclampsia, and intrauterine growth restriction (IUGR) (Bolatai et al. 2022). In these adverse pregnancy conditions, differences of VEGFA and PlGF concentrations in the maternal serum, as well as disturbances in placental expression, were observed (Burton et al. 2009). Fibroblast growth factor 2 (FGF2), a leading member of the FGF family of heparin-binding growth factors, contributes to normal as well as pathological angiogenesis (Jia et al. 2021). In the process of normal embryogenesis and the development of pregnancy after in vitro fertilization, proper pro-angiogenic stimulation of vascular growth and antiangiogenic inhibition of vascular hypertrophy are of particular importance (Huppertz and Peeters 2005; Burton et al. 2009; Chen and Zheng 2014; Bhattacharjee et al. 2021).

To understand the mechanisms underlying adverse pregnancy outcomes, biomarkers of placental dysfunction are sought. A well-functioning placenta is crucial for the course of pregnancy and health of the future child. Understanding the differences at the molecular level between normal placentas and placentas after in vitro fertilization may allow the improvement of assisted reproductive techniques. Gene expression in the placenta could be influenced by many factors including fetal gender, gestational age, or mode of delivery. Variation in gene expression may also occur in different regions of the placenta due to differences in placental architecture or blood supply, and therefore, consistency in placental sampling is of key importance (Burton et al. 2014; Janssen et al. 2015).

The main aim of this case-control study was to determine mRNA expression of angiogenesis-related genes (*VEGFA*, *FGF2*, *KDR*, and *FLT1*) in placentas of women after assisted reproductive technology fertilization versus fertile women with uncomplicated pregnancy. We also analyzed the differences in placental expression of these genes depending on the type of embryo transfer (fresh/frozen), place of tissue donation (center/margin), and newborns’ gender (male/female).

## Materials and methods

### Material

The study was conducted in the Department of Infertility and Reproductive Endocrinology of Poznan University of Medical Sciences, Poznan, Poland, between January 2020 and June 2022, in a group of Caucasian women with singleton pregnancy. The samples from 29 placentas were collected. Anthropomorphic data of mothers and their newborns, laboratory results, and information on the course of pregnancy were obtained from medical records. This *case-control study* consisting of 20 infertile women who underwent an ART treatment cycle and 9 healthy women as a control group without evidence of reproductive difficulty had naturally conceived pregnancies. Patients and controls were of Polish origin, from the same geographical area.

Exclusion criteria for the study group were as follows: multiple pregnancies, an abnormal karyotype of parents and any inborn defects or chromosomal abnormalities, maternal age below 18 years, systemic connective tissue disorder, antiphospholipid antibody syndrome, hereditary thrombophilia, positive antinuclear antibodies, endocrine dysfunction (luteal insufficiency, hyperprolactinemia, thyroid diseases), and an alternative reason for subfertility such as infectious and anatomical causes, cigarette smoking, or alcohol drinking. All infertile women received luteal phase support and underwent intracytoplasmic sperm injection to increase the chance of conception and had their own good quality embryos available for transfer. Also, women in the study group had a regular menstrual cycle and an optimal basal serum follicle-stimulating hormone (FSH) level measured on the third day of the last cycle. None of the patients had been taking hormone therapy within the last 3 months. Fresh or frozen embryo transfer was always performed on the fifth day, by two people (minimum 15 years of experience in the same clinic). Preimplantation genetic screening and diagnosis (PGS/PGD) methods were not applied due to the lack of medical indications.

Women from the control group had regular menstrual cycles, no evidence of autoimmunity, no past history of pregnancy loss, and no gestational complications or immunological and endocrine diseases. Multiple pregnancies, maternal age below 18 years, cigarette smoking, and alcohol drinking were also criteria for exclusion from the control group.

The research study was approved by the Ethics Committee of the Poznan University of Medical Sciences (no. 1159/19, date: 5 December 2019). Prior to delivery, women provided written informed consent for the research studies conducted on their placental tissue collected after delivery.

### Methods

#### Tissue collection

All placental samples were taken immediately after caesarian section delivery by one person present at all deliveries (MA). Placental tissues were collected at the center (close to the cord insertion) and margin (distal edge) part of the placenta avoiding the hemorrhage, necrosis, and calcification areas. After washing the blood, the placental samples were processed in approximately 1 cm^3^ fragments and placed in tubes with an appropriate amount of Invitrogen RNAlater Stabilization Solution (Invitrogen, Waltham, Massachusetts, USA) and stored at – 80 °C until RNA extraction.

#### RNA isolation, cDNA synthesis, and quality control

RNA isolation and real-time quantitative PCR (RT-qPCR) were conducted in the Institute of Human Genetics, Polish Academy of Sciences, Poznan, Poland. Placenta samples were homogenized with an electric homogenizer and subjected to RNA isolation with TRIzol Reagent (Life Technologies, Carlsbad, California), according to the manufacturer’s procedure. For all obtained RNA samples, a quantitative and qualitative evaluation was carried out using an Agilent RNA 6000 Nano Kit and the Bioanalyzer 2.0 equipment (Agilent, Santa Clara, CA, USA). Two micrograms of total RNA with RIN ≥ 7 was converted to cDNA with an iScript Advanced Reverse Reaction kit (Bio-Rad) with the following conditions: 25 °C for 5 min–annealing step; 42 °C for 30 min–reverse transcription; and 95 °C for 1 min–inactivation.

#### Real-time quantitative PCR

For the study, four angiogenesis-related genes were selected: vascular endothelial growth factor A (*VEGFA*, gene ID 7422), fibroblast growth factor 2 (*FGF2*, gene ID 2247), fms-related receptor tyrosine kinase 1 (*FLT1*, gene ID 2321), and kinase insert domain receptor (*KDR*, gene ID 3791)

The mRNA levels of *VEGFA*, *FGF2*, *KDR*, and *FLT1* genes were measured by RT-qPCR on a Bio-Rad CFX Connect 96-well Thermal Cycler (Bio-Rad Laboratories, Inc., Hercules, California, USA) using the iTaq Universal SYBR Green Assay (Bio-Rad Laboratories, Inc., Hercules, California, USA), according to the manufacturer’s instructions. Primers for analysis were designed using the Primer-BLAST tool, and their sequences are presented in Table 1. Primers for the reference *ACTB* gene were ordered as PrimePCR SYBR Green Assay by Bio-Rad Laboratories, Inc. Every reaction was performed in duplicate. The threshold cycle (CT) values of all samples were recorded, and relative fold changes (FC) in gene expression were analyzed using the 2^−ΔΔCT^ method (Livak and Schmittgen 2001).

**Table 1:** **Se**quences of primers used in real-time quantitative PCR

Gene	5′-3′ sequence	Amplicon size (bp)	Annealing temp. (°C)
*VEGFA*	TGCAGATTATGCGGATCAAACC	81	60
	TGCATTCACATTTGTTGTGCTGTAG		
*FGF2*	AGAGCGACCCTCACATCAAG	224	60
	TCGTTTCAGTGCCACATACC		
*KDR*	TGGTATTGGCAGTTGGAGGAAG	83	59
	CATTCTTCACAAGGGTATGGGTTT		
*FLT1*	TGGCAGCGAGAAACATTCTTTTATC	200	59
	CAGCAATACTCCGTAAGACCACAC		

### Statistical analysis

The R statistical software version 4.3.1 (R Foundation for Statistical Computing, Vienna, Austria, accessed on 25 June 2023) was used for statistical analysis. The ggplot2 (version 3.4.3) and ggstatplot (version 0.12.1) packages were used for data plotting (Patil 2021; Wickham 2016). The normal distribution was tested using the Shapiro-Wilk test. Quantitative variables with normal distribution were presented as mean ± standard deviation (SD) and in the absence of Gaussian distribution as median and interquartile range (IQR). For nominal variables, the chi-square or Fisher’s test was used. RT-PCR data were processed based on the ∆∆Ct model using the PCR R package (version 1.2.2) (Ahmed and Kim 2018) and the data were expressed as the mean value (± SD). Statistical significance between two groups was determined by Student’s *t*-test. One-way analysis of variance (ANOVA) was used to compare three groups, followed by Tukey’s multiple comparison test. Associations between the expression of genes were assessed using the nonparametric Spearman rank correlation test with Holm-Bonferroni correction. A *p*-value less than 0.05 was considered to indicate statistical significance.

## Results

### Subjects’ characteristics

Baseline characteristics of the study population are shown in Table 2. The study included 29 women from whom placental samples were collected after delivery. Among these, 9 women gave birth to a child after natural conception (NC) as controls, 10 after in vitro fertilization in a fresh cycle (ET), and 10 after a frozen cycle (FET). All 29 pregnancies were terminated by cesarean sections. The analyzed data of mothers and their newborns did not show statistically significant differences between the groups. The highest average placental weight was recorded in the FET group 695.00 ± 89.60 g vs. 594.44 ± 166.29 g in ET and 580.00 ± 53.18 g in NC (*p* = 0.080). Frozen embryo transfer resulted in the highest birth weight (3679.00 ± 273.36 g) compared to the controls (3500.63 ± 408.89 g, *p* = 0.634) and fresh ET (3274.44 ± 522.31 g, *p* = 0.101). In both IVF groups, the numbers of oocytes retrieved and transferred embryos were comparable. No statistically significant differences were observed when comparing hormone concentrations between the fresh and f*rozen embryo transfer* groups.

**Table 2:** Comparison of obstetrical and neonatal parameters in study groups.

Characteristics	Controls (*N* = 9)	ET (*N* = 10)	FET (*N* = 10)	*p*
Age (years), mean ± SD	31.38 ± 4.75	34.89 ± 5.01	33.70 ± 4.67	0.330
Weight (kg), mean ± SD	70.00 ± 9.46	65.00 ± 9.96	66.50 ± 13.36	0.650
Height (cm), mean ± SD	168.25 ± 6.65	169.22 ± 6.70	166.20 ± 4.94	0.561
BMI (kg/m^2^), mean ± SD	24.94 ± 4.65	22.69 ± 3.04	24.09 ± 4.88	0.553
Gestational age (weeks), mean ± SD	39.00 ± 0.93	38.33 ± 1.50	39.20 ± 1.40	0.350
Parity, *n* (%)				
Primigravida	5 (55.56)	2 (20.00)	3 (30.00)	0.248
Multigravida	4 (44.44)	8 (80.00)	7 (70.00)	
Birth weight (g), mean ± SD	3500.63 ± 408.89	3274.44 ± 522.31	3679.00 ± 273.36	0.121
Placenta weight, (g), mean ± SD	580.00 ± 53.18	594.44 ± 166.29	695.00 ± 89.60	0.080
Newborn sex, *n* (%)				
Male	7 (77.80)	4 (40.00)	6 (60.00)	0.247
Female	2 (22.20)	6 (60.00)	4 (40.00)	
Apgar score, median [IQR]				
At 5 min	10 [10; 10]	10 [10; 10]	10 [10; 10]	0.903
At 10 min	10 [10; 10]	10 [10; 10]	10 [10; 10]	0.575
pH of blood at delivery, median [IQR]				
Artery pH	7.24 [7.18; 7.28]	7.27 [7.20; 7.32]	7.28 [7.26; 7.30]	0.343
Vein pH	7.33 [7.27; 7.37]	7.33 [7.30; 7.34]	7.34 [7.28; 7.36]	0.861

### The mRNA expression levels in the marginal and central parts of the placenta

**Fig. 1:**
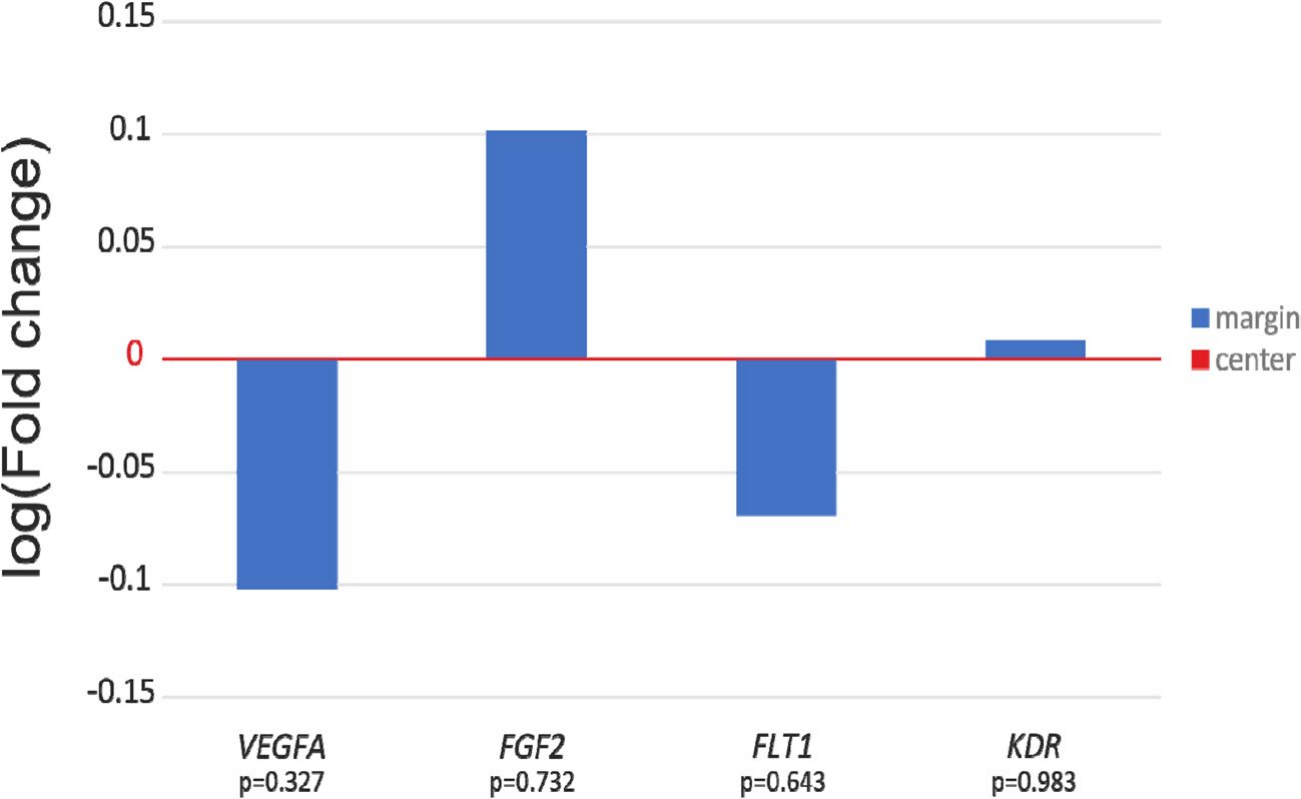
Relative expression of angiogenesis-related genes in samples taken from central and margin areas of placentas. Log mean fold gene expression is shown. Differences in gene expression were analyzed using the independent samples *t*-test

Comparing the average ∆Ct of the tested genes in the marginal and central parts of the placenta for the whole group (*N* = 29), no statistically significant differences were observed. As shown in Fig. 1, the placental relative mRNA expression of the *VEGFA* and *FLT1* genes was 0.79 and 0.85-fold lower in margin samples compared to central sampling sites (*p* = 0.327 and *p* = 0.643, respectively). The *KDR* gene expression in the marginal part was almost identical to that in the central part (FC = 1.02, *p* = 0.983), while the expression of the gene *FGF2* was slightly up-regulated in the margin part of placentas (FC = 1.26, *p* = 0.732).

### Placental mRNA expression in IVF and control groups

Since the placental tissue sampling site did not significantly affect the expression of the studied genes, we presented the results for the average values from the central and marginal parts of the placenta in further analysis.

**Fig. 2:**
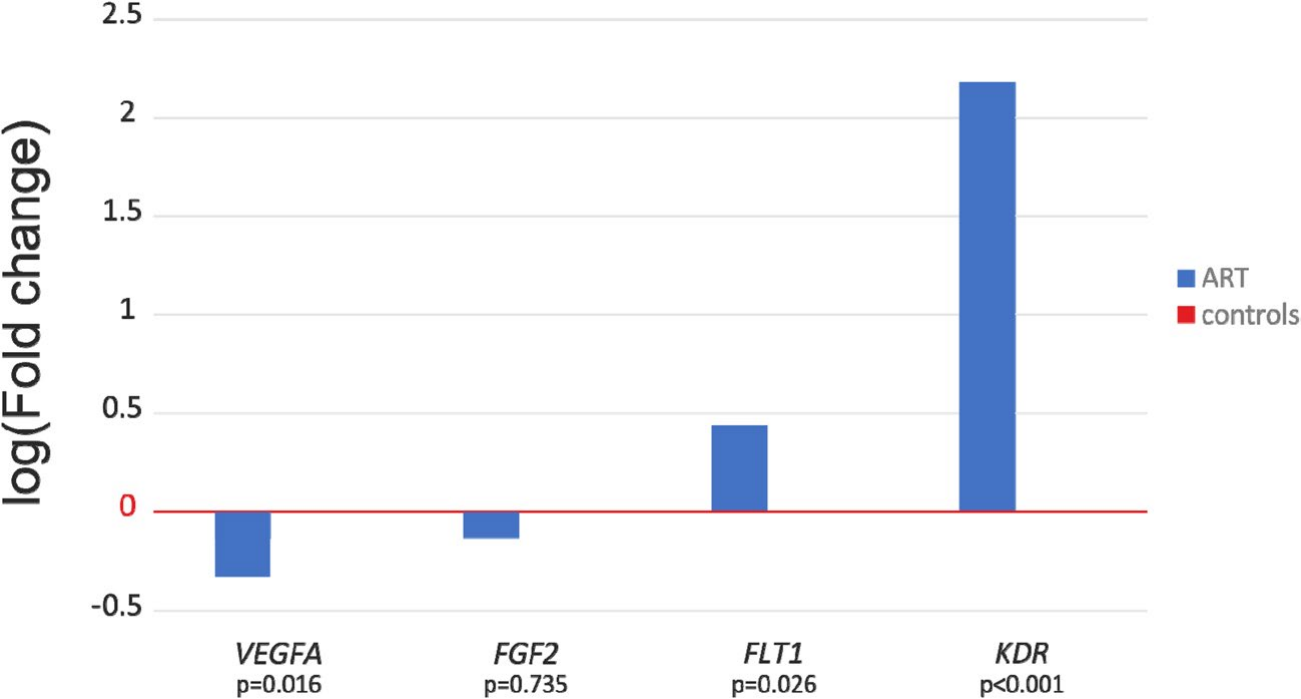
Angiogenesis-related genes’ placental relative expression in cases and controls. Log mean fold gene expression is shown. Differences in gene expression were analyzed using the independent samples *t*-test

**Fig. 3:**
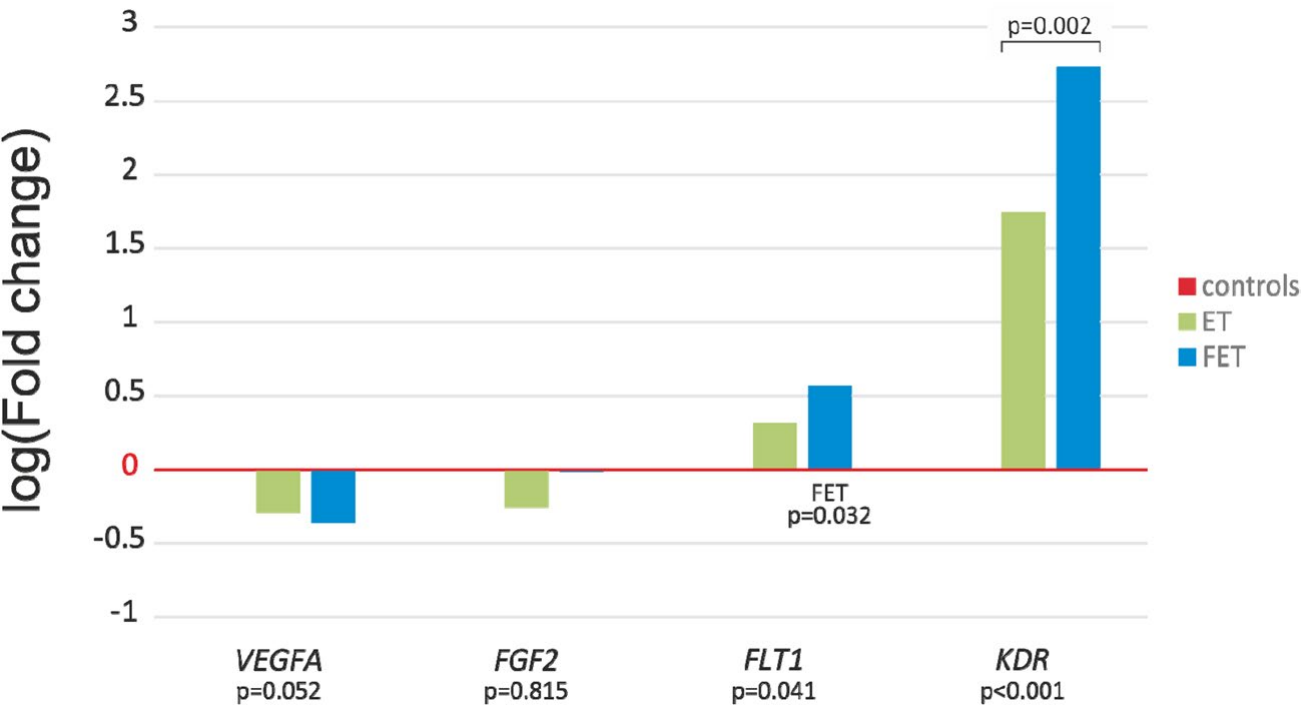
Angiogenesis-related genes’ placental relative expression in fresh and frozen *embryo transfer*. Log mean fold gene expression is shown. Differences in gene expression were analyzed using the independent samples *t*-test or ANOVA with Tukey’s post hoc test

In the placentas of mothers who used assisted reproduction technologies, significant down-regulation of the *VEGFA* gene (FC = 0.47, *p* = 0.016) was observed (Fig. 2). Interestingly, statistically significant up-regulation was observed for both *VEGFA* receptors *FLT1* and *KDR* (*p* = 0.026 and *p* < 0.001 respectively) (Fig. 3).

### The relationship between the type of transformed embryo and placental mRNA expression

The relative mRNA levels of studied angiogenesis-related genes among ET, FET, and control groups were also compared by one-way ANOVA, followed by Tukey’s post hoc test. Comparing placental gene expression in these three groups, no statistically significant differences were found for *VEGFA* (*p* = 0.052 in ANOVA) and *FGF2* (*p* = 0.815 in ANOVA). Both genes showed lower expression compared to controls in both the ET and FET groups.

Genes encoding VEGFA receptors were up-regulated in both groups compared to controls, and the FET group had the highest relative expression values. For the *FLT1* gene, a statistically significant difference was observed (*p* = 0.041 in ANOVA). The post hoc test showed significant differences between the frozen embryo transfer group and the controls (*p* = 0.032). Placental expression of the *KDR* gene was statistically significantly different between all analyzed groups (for both embryo transfer groups and controls, *p*-values were < 0.001, and between ET and FET, the *p*-value was 0.002 in the post hoc test) (Fig. 3).

### Association of newborn gender with placental mRNA expression

**Fig. 4:**
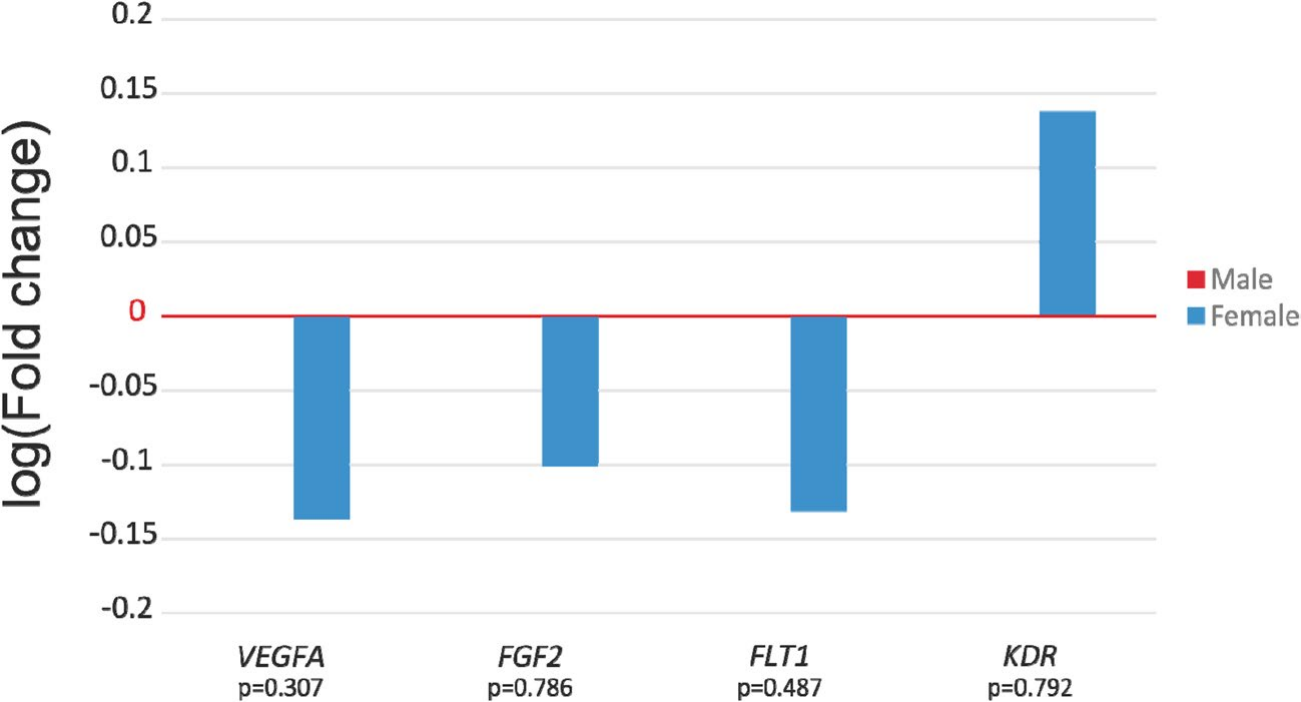
Angiogenesis-related genes’ placental relative expression in newborn gender groups. Log mean fold gene expression is shown. Differences in gene expression were analyzed using the independent samples *t*-test

Comparing gene expression in male (*N* = 17) and female (*N* = 12) neonates, there was no difference in *VEGFA* (*p* = 0.307), *FGF2* (*p* = 0.786), *FLT1* (*p* = 0.487) or *KDR* mRNA expression (*p* = 0.792), which indicates that gender does not affect placental expression of angiogenesis-related genes. In female neonates, the expression of *VEGFA* (FC = 0.73), *FGF2* (FC = 0.79) and *FLT1* (FC = 0.74) was reduced compared to male neonates. Only for the *KDR* gene did we observe higher expression in female newborns (FC = 1.37) (Fig. 4).

### Correlation between mRNA expression levels in placenta

**Fig. 5:**
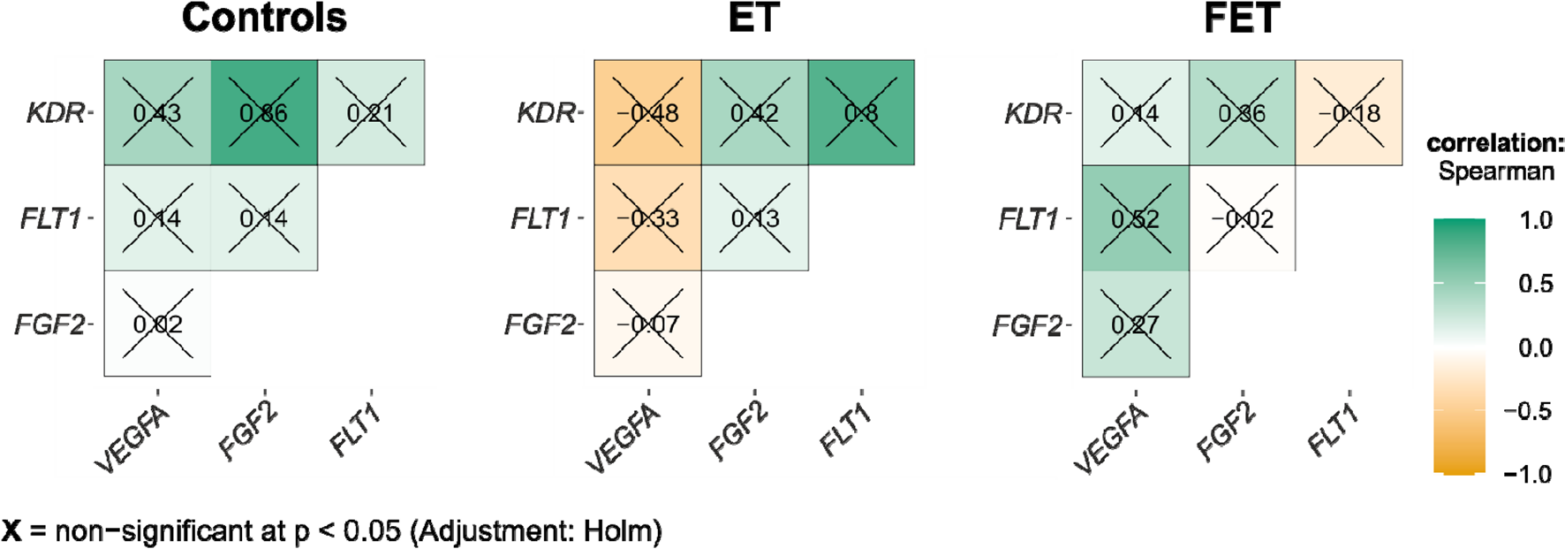
Correlations between *VEGFA*, *FGF2*, *FLT1*, and *KDR* gene expression in control, ET, and FET groups

The results of the statistical analysis did not confirm the occurrence of statistically significant correlations, either between the tested genes or between gene expression levels and clinical data of newborns. The observed associations in the control, ET, and FET groups are presented in Fig. 5. The strongest correlation observed in groups were for controls between *KDR* and *FGF2* genes (rho = 0.86, *p* = 0.014, *p*_corr_ = 0.082), for ET between *FLT1* and *KDR* (rho = 0.80, *p* = 0.010, *p*_corr_ = 0.058), and for FET between *VEGFA* and *FLT1* (rho = 0.52, *p* = 0.183, *p*_corr_ > 0.999).

## Discussion

Despite the dynamic progress of molecular research, many mechanisms influencing the proper development of the placenta remain unexplained. Many authors have focused on pathways related to vascular angiogenesis in the uteroplacental circulation. Deserving our particular attention should be possible changes in the expression of angiogenic factors in the process of fetal development, after embryo transfer in the IVF procedure. Even subtle variations can cause a negative IVF result or affect the newborn’s health. It is important to identify any modifiable elements in the IVF procedure to optimize outcomes for parents and their offspring. Special attention is paid to possible changes in the expression of angiogenic factors and their receptors in the process of embryo development after ET/FET administration.

Placental gene expression during pregnancy is constantly changing, adapting the immune response to the need for tolerance, and modifying metabolism according to the demands of pregnancy. It was also reported that in some pathological conditions during pregnancy, under the influence of hypoxia/ischemia, changes in expression patterns of VEGF, PlGF, and their receptors FLT-1/KDR in the placenta were noted. Kumazaki et al. observed in the regions of hypoxia/ischemia increased expression of VEGF and KDR in the vessels of the villi (42 samples of placentas from normal and complicated pregnancies ended in delivery in the 2nd/3rd trimester). Additionally, there was increased expression of PlGF and FLT-1 in the cells of the villous trophoblast. An interesting observation was that VEGF and PlGF stimulated the migration of inflammatory cells through autocrine regulation of the FLT-1 receptor in the placenta. Thus, the VEGF family has multiple functions and the changes of its expression are actively involved in the development of pathological features in the placenta (Kumazaki et al. 2002).

Altered expression of angiogenic factors and its receptor both in serum and placenta was also detected in some pregnancy complications. In a study performed by Meng et al., it was found that expression of the angiogenic factor VEGFA and its receptor VEGFR2 mRNA and protein was significantly reduced in the gestational diabetes mellitus (GDM) group (*p* < 0.05) compared to the expression in placentas from normal pregnancies (Meng et al. 2016). Troncoso et al. reported that GDM was associated with reduced expression levels of FLT-1 and KDR mRNA, but higher levels of KDR protein in the placenta (Troncoso et al. 2017). In another study, decreased mRNA expression of VEGFA, PlGF, KDR, and FLT1 was found in preeclamptic preterm placentas. Decreased expression of VEGFA, PGF, and KDR mRNA in placentas from pregnancies complicated by gestational hypertension was also observed, as well as reduced expression of VEGFA mRNA in placentas from pregnancies complicated by SGA with normal pressure (Andraweera et al. 2012). In contrast, Ahmed et al. found that the expression of PIGF mRNA and protein was higher in IUGR placentas compared to placentas from normal pregnancies (Ahmed et al. 2000). However, the results are not clear. Lash et al. detected no differences in the expression of any of the VEGF isoforms in the placentas from pregnancies complicated by IUGR and fetal macrosomia and in the placentas of normal pregnancies (Lash et al. 2001).

Andraweera et al. summarized the role of angiogenic growth factors in the pathophysiology of some pregnancy complications, indicating the significance of VEGF family biomarkers, especially PlGF and FLT-1 factors, in the prediction of early-onset preeclampsia. These markers, measured in the late first or second trimester of pregnancy, predict early-onset preeclampsia with high sensitivity and specificity (Andraweera et al. 2012).

There is a limited understanding of possible placental expression modifications of angiogenesis-related genes occurring after transfers of both fresh and frozen embryos in the IVF procedure. To the best of our knowledge, this study is the first to analyze the placental expression of angiogenic factors in a population of Polish women undergoing an IVF procedure. We observed significant differences in the mRNA expression of *VEGFA*, *FLT1*, and *KDR* genes in the placentas of women who became pregnant after embryo transfer, compared to the control group. Despite the lack of statistically significant differences in placental expression of *VEGFA* and *FGF2* genes, between the three studied groups, their lower expression in both ET and FET groups compared to the controls should be noted. The most interesting results were obtained by analyzing placental mRNA expression of VEGF receptors. The expression of the *KDR* gene was statistically significantly different between all analyzed groups, whereas the *FLT1* gene varied only between the FET and the controls. These results could elucidate, at least partially, which of the angiogenic factors are the most important for balanced placenta development after embryo transfer in the IVF procedure.

In our study, no significant differences in placental mRNA expression of *VEGFA*, *FGF2*, *FLT1*, and *KDR* genes depending on the sampling site were observed. Similarly, Chinni et al. (2008) found that placental expression of genes related to hemostasis and angiogenesis, involving placental VEGF expression, was not dependent on the method of sampling (Chinni et al. 2008). In contrast, in the study of Yong et al. focusing on fetal growth retardation (FGR), significantly lower VEGF expression in the peripheral area of the placenta compared to the central part was recorded. Additionally, the lower VEGF expression was correlated with pathological features in the FGR placenta (changes in terminal villi, trophoblast, and capillary volume). The authors suggested that at least one peripheral and central sample should be taken to assess placental expression of analyzed factors. This could enable better clinical interpretation of the results and demonstrate that the placental sampling technique alone can improve the reproducibility of the results (Yong et al. 2023).

In our research, we also focused on the interesting question of possible differences between female and male fetuses in expression of placental genes involved in angiogenesis. Further analysis did not reveal any statistically significant differences depending on gender in our studied groups. Nevertheless, several studies have reported differences in gene and protein expression levels between the placentas of male and female fetuses. Jiang et al. analyzed the placental *VEGFA*, *VEGFC*, *ANGPT1*, and *ANGPT2* genes’ expression involved in angiogenesis. These genes showed higher expression in placentas from female compared to male fetuses (Jiang et al. 2020). Differential expression of placental miRNAs implicated in adipogenesis has also been observed in placentas collected from female but not male fetuses (Tsamou et al. 2017). These differences between placentas from female and male fetuses in terms of angiogenesis, nutrient transport, and immune and inflammatory responses could implicate the risk of development of pregnancy complications (Aspritoiu et al. 2021).

Many studies have shown that the frozen embryo transfer cycle may affect the birthweight of the newborn (Maheshwari et al. 2018; Laval et al. 2020; Woo et al. 2017). Probably high estrogen and progesterone concentrations from controlled ovarian stimulation during fresh ET may affect genes involved in implantation, impaired early implantation, placentation, and subsequent fetal growth. Furthermore, high estrogen levels during ovarian stimulation have been postulated to interfere with endometrial angiogenesis (Pereira et al. 2017; Kalra et al. 2011; Yang et al. 2022). In our study, we also observed a higher, but not significantly, birth weight in the FET group compared to the ET group, which may confirm that the type of embryo transfer may affect the birth weight of the newborn. On the other hand, in our group of frozen embryo transfers, delivery was slightly later and more boys were born, which may have contributed to the increase in birth weight in this group.

In our research, the number of placental samples is relatively small, which is the result of accurate selection of women to the study groups. Especially, the exclusion criteria were chosen carefully to select appropriate patients. Nevertheless, the number of specimens in our study is comparable to the other researches that presented the similar results (Zhao et al. 2020; Chen et al. 2015; Gutman et al. 2008).

Our study showed no statistically significant differences in perinatal outcomes between investigated groups of fresh and frozen embryos. However, we observed the significant difference in placental expression of KDR gene between ET and FET groups which suggests that the changes in angiogenesis could be involved in placenta development and fetal growth. This relationship could potentially find an application in therapy of couple’s infertility but it should be confirmed in the larger sample of patients. However, similar observations are reason that currently, emphasis is placed on of the freeze-all policy procedure, in which frozen embryos are used for transfer in order to reduce the percentage of pregnancy complications.

## Summary

Our results expand the current knowledge about the function of genes related to placental angiogenesis in pregnancies after assisted reproductive technologies. We documented differences in the expression of the *VEGFA* gene and its receptors FLT1 and KDR in placentas after transfer of both fresh and frozen embryos, compared to the group after natural conception. These differences could suggest the important role of angiogenic factors in placenta development in both fresh and frozen embryo transfers. In the case of the genes examined in this study, their placental mRNA expression was not dependent on the place of tissue collection from the placenta or on the fetus gender. Researching the differences in the expression of genes involved in angiogenesis will allow better understanding of key biological pathways regulating the proper functioning of the placenta and fetal development. These observations certainly need to be expanded and confirmed in future studies. However, the presented variations in the process of placental angiogenesis depending on the transformed embryo may be used to improve assisted reproductive technologies.
